# Case Report: Non-bacterial thrombotic endocarditis and multiple thrombi uncover a hidden prothrombotic mutation

**DOI:** 10.3389/fcvm.2026.1775477

**Published:** 2026-07-07

**Authors:** Yongchao Luo, Lanlin Zhang, Xiang Zhang, Guotao Ma

**Affiliations:** 1 Department of Breast Surgery, Peking Union Medical College Hospital, Chinese Academy of Medical Science and Peking Union Medical College, Beijing, China; 2 Department of Cardiac Surgery, Peking Union Medical College Hospital, Chinese Academy of Medical Science and Peking Union Medical College, Beijing, China; 3 Department of Pathology, Peking Union Medical College Hospital, Chinese Academy of Medical Science and Peking Union Medical College, Beijing, China

**Keywords:** case report, F2 gene, hereditary thrombophilia, non-bacterial thrombotic endocarditis, tricuspid valve vegetation

## Abstract

**Background:**

Non-bacterial thrombotic endocarditis (NBTE) is a rare, sterile valvular condition associated with hypercoagulable states, malignancy, or autoimmune diseases. Diagnosis is challenging due to nonspecific symptoms and unknown cause.

**Case presentation:**

A 14-year-old male presented with acute-onset severe abdominal pain. Imaging studies revealed extensive thrombi involving multiple organ veins, as well as a large vegetation on the tricuspid valve, consistent with NBTE. Although routine thrombophilia screening was unremarkable, a significant family history of thrombotic events prompted further genetic evaluation. Whole-exome sequencing identified a pathogenic heterozygous variant in the *F2* gene (*F2* c.1621C > T, p.Arg541Trp), confirming an underlying hereditary thrombophilia. The patient underwent successful surgical excision of the vegetation with tricuspid valve repair. Anticoagulation therapy resulted in complete resolution of symptoms, and long-term management was initiated to mitigate recurrent thrombosis risk.

**Outcome:**

At one-year follow-up, repeat imaging showed recanalization of the portal and splenic veins without recurrent thrombosis, underscoring the effectiveness of anticoagulation.

**Conclusion:**

This case demonstrates an atypical NBTE presentation with tricuspid valve involvement and extensive venous thrombosis due to a rare *F2* mutation. The successful treatment reflects the importance of early recognition, multidisciplinary management and detailed consultation.

## Introduction

Non-bacterial thrombotic endocarditis (NBTE) is a rare and often underdiagnosed form of sterile endocardial inflammation, typically involving cardiac valves and occurring in patients with underlying systemic conditions (1). It more commonly affects the mitral and aortic valves, followed by the tricuspid and pulmonary valves (2, 3). Previous studies have shown that the occurrence of NBTE is linked to cancer, immune diseases, and hypercoagulable states (4–6) NBTE occasionally presents with chest pain, dyspnea, and, rarely, cardiac murmurs, but it remains clinically silent most of the time (5). It carries a high risk of severe complications, most notably systemic embolization, which most commonly presents as cerebral infarction, followed by infarctions of the kidneys, spleen, lungs, and myocardium (7).

The diagnosis of NBTE is particularly challenging and requires careful differentiation from infective endocarditis (IE), guided by the modified Duke criteria. Patients with NBTE typically have negative blood cultures, no evidence of endocardial injury, and absence of fever (1, 5). As with IE, NBTE is recommended to be initially evaluated with transthoracic echocardiography (TTE), which can detect nearly half of all valvular vegetations. If TTE is negative, transesophageal echocardiography (TOE) may be performed, as it offers higher sensitivity and can detect vegetations smaller than 5 mm (2, 8). CT and magnetic resonance imaging (MRI) may also aid in the identification of NBTE. Currently, there are no established guidelines for the treatment of NBTE. However, anticoagulation and management of the underlying condition—such as malignancy or autoimmune disease—are generally accepted approaches (5, 9). Surgical intervention may be considered on a case-by-case basis, depending on factors such as the severity of valvular regurgitation and the size of the vegetation (1).

Thrombophilia is a complex pathological condition characterized by an increased tendency toward thrombosis. It can be broadly classified into acquired and inherited forms. Acquired thrombophilia is exemplified by antiphospholipid antibody syndrome (APS) (10) and is also associated with other conditions such as cancer (11). Inherited thrombophilia represents a genetic predisposition to thrombosis (12), commonly involving inactivating mutations in genes responsible for natural anticoagulants—antithrombin (AT), protein C (PC), and protein S (PS)—as well as activating mutations in factor Ⅴ (e.g., factor Ⅴ Leiden, FⅤL) and prothrombin (F Ⅱ) (*F2* c.97G > A, formerly known as G20210A) (13). Besides the conventional variants described above, several rare genetic mutations have been implicated as possible contributors to thrombophilia (14, 15).

This report presents a rare case of NBTE involving the tricuspid valve in a previously healthy adolescent male, with extensive venous thromboses and an underlying rare *F2* gene mutation. It highlights the diagnostic challenges, clinical course, and significance of thorough family history and genetic testing in identifying inherited thrombophilia.

## Case description

**Figure 1:**
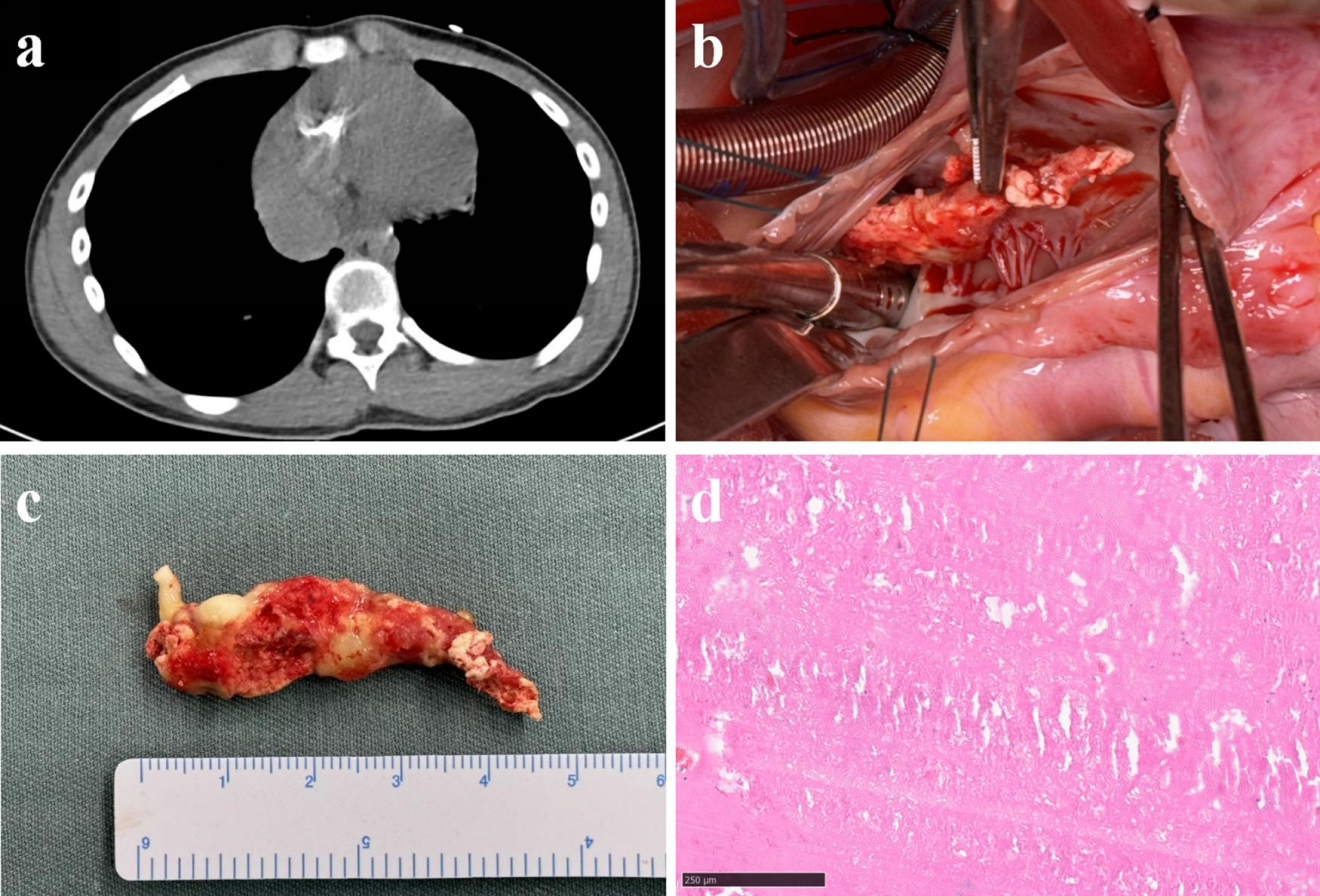
**(a)** tricuspid valve vegetation with calcification on CT. **(b,c)** Gross aspect of tricuspid valve vegetation. **(d)** Histology showed vegetations to be composed almost entirely of ﬁbrin. Scale bar =250 μm.

A 14-year-old male with no significant medical history awoke with the sudden onset of diffuse, dull, and distending abdominal pain (12 days before surgery). The pain was non-radiating and unrelated to food intake. He reported no fever, chills, dizziness, syncope, diarrhea, hematochezia, palpitations, or dyspnea and had no history of recent infection, trauma, or immobilization. He initially self-treated with herbal remedies and was discharged with a proton pump inhibitor (PPI) at a local hospital. However, his symptoms persisted and it prompted referral to a regional medical center for further evaluation. At the referring hospital, contrast-enhanced abdominal computed tomography (CT) demonstrated extensive thrombus formation involving the mesenteric, splenic, and portal venous systems. Given this unusual thrombotic burden in a healthy adolescent, a broader workup was initiated. Chest CT revealed bilateral segmental pulmonary emboli localized to the lower lobes. Transthoracic echocardiography revealed a 45-mm-long mobile mass on the tricuspid valve, with moderate right ventricular dilation and severe tricuspid regurgitation (4 days before surgery). Blood cultures were obtained and returned negative. Given the risk of embolization and tricuspid valve mass progression, the patient was referred to our institution for comprehensive evaluation and management. Repeat CT pulmonary angiography confirmed bilateral emboli and tricuspid valve mass (Figure 1a), and on physical examination, the patient was afebrile, with no jugular venous distention. Cardiac auscultation also revealed a systolic murmur over the tricuspid valve area. The abdomen was nondistended, without tenderness, rebound tenderness, or guarding, and there was no hepatomegaly. No lower extremity swelling or varicosities were observed, and bilateral lower limb skin temperature was normal and symmetric. No Janeway lesions or Osler nodes were noted on the extremities. A multidisciplinary team meeting, including specialists from cardiothoracic surgery, cardiology, hematology, rheumatology and immunology, and infectious diseases, was held. Although systemic anticoagulation is generally the cornerstone of therapy for NBTE, surgery was selected in this case after multidisciplinary evaluation. The rationale included the presence of a large tricuspid vegetation with concern for potential embolic risk, as well as the need for definitive pathological diagnosis and exclusion of infective endocarditis. Therefore, the patient was transferred to the cardiothoracic surgery department for vegetation excision and valve repair under cardiopulmonary bypass. Intraoperative exploration revealed an irregular, columnar mass measuring approximately 45 mm at its greatest dimension, attached to the anterior leaflet of the tricuspid valve, its subvalvular chordae, and the right ventricular wall (Figure 1b). Grossly, the lesion was yellowish and friable with a calcified base, consistent with an organized thrombus (Figure 1c). After surgery and a period of observation in the intensive care unit, the patient was transferred to the general ward of the cardiothoracic surgery department. Postoperative recovery was uneventful, and the patient's abdominal pain gradually subsided with continued anticoagulation therapy.

After being dischargedl, we instructed the patient to take 4.5 mg of warfarin for six months, with the goal of maintaining the INR between 2.0 and 3.0. Follow-up (1 year after surgery) imaging in the outpatient setting revealed recanalization of the portal and splenic veins after thrombosis, further supporting the effectiveness of anticoagulant therapy.

**Figure 2:**
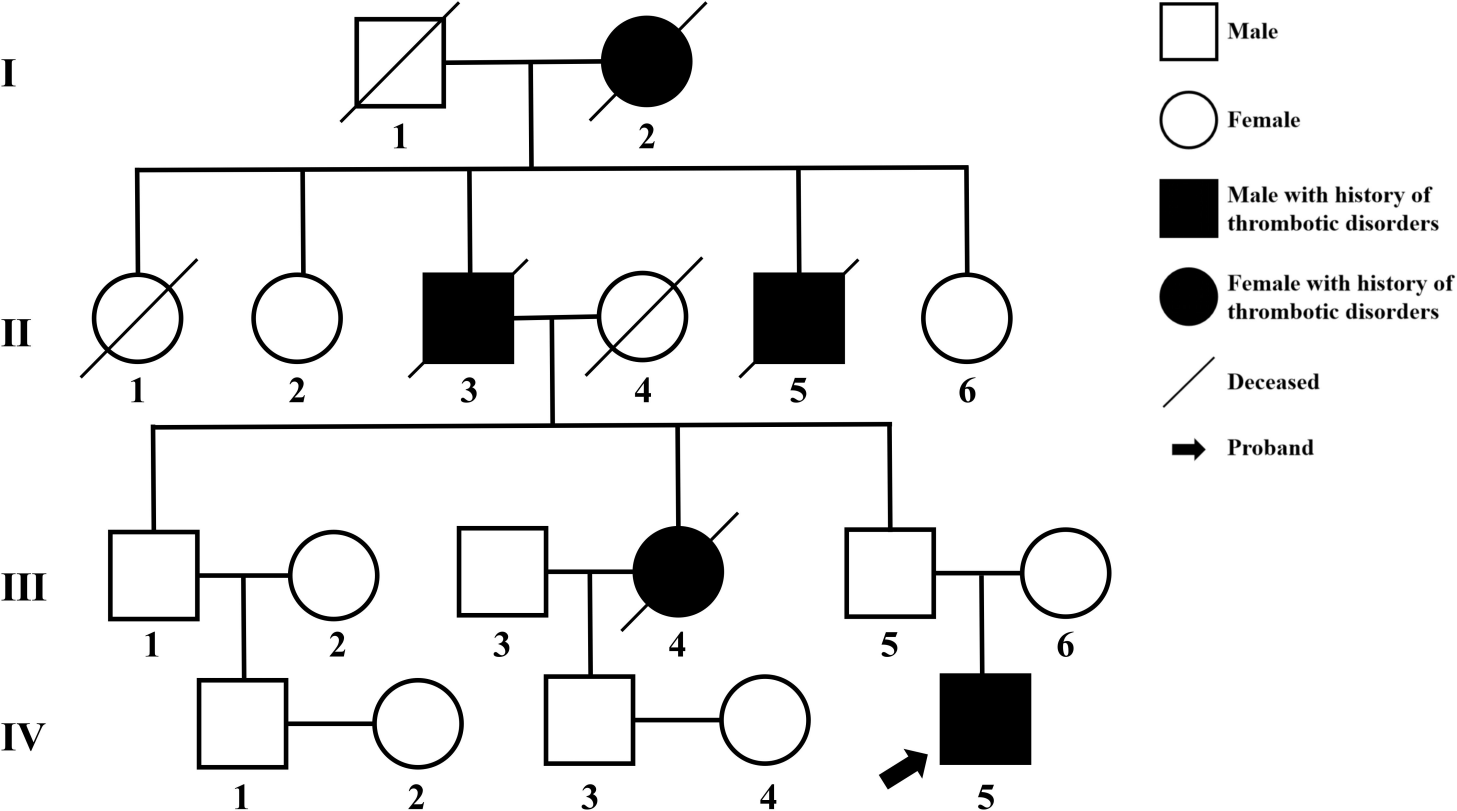
Pedigree of a four-generation family with thrombotic disorders. Filled symbols represent affected individuals, open symbols represent unaffected individuals, squares represent males, and circles represent females. Diagonal lines indicate deceased individuals. The proband is indicated by an arrow.

Histopathological examination demonstrated vegetations to be composed almost entirely of fibrin (Figure 1d). It favored a diagnosis of NBTE over infectious endocarditis. Therefore, further laboratory testing was performed to investigate the underlying cause of the valvular thrombotic vegetation and the multiple thrombi at other sites, all of which yielded negative results. Conventional thrombophilia, autoimmune, and antiphospholipid antibody screening was largely unremarkable, whereas genetic testing identified a likely pathogenic heterozygous F2 variant, c.1621C > T (p.R541W), in both the patient and his father (Supplementary S1–S3**)**. A detailed family history revealed that several of the patient's relatives had a history of thrombotic disorders, raising a strong suspicion for an inherited thrombophilia (Figure 2). Finally, genetic analysis identified an F2 gene variant, c.1621C > T, in both the patient and his father. Based on the patient's clinical manifestations, laboratory findings, pathological results, and treatment response, he was ultimately diagnosed with NBTE and multiple thrombotic events in the setting of inherited thrombophilia, with the F2 variant considered a possible contributing factor. The clinical timeline is summarized in Supplementary S4.

## Discussion

This case illustrates an unusual presentation of NBTE in a young patient, with unique involvement of the tricuspid valve and extensive venous thrombosis. The diagnostic challenge stemmed from the initial nonspecific symptom of abdominal pain and the absence of classic signs of infective endocarditis (fever, positive blood cultures, and systemic infection). The diagnosis of NBTE was supported by echocardiography, negative cultures (Two sets of blood cultures remained negative after 7 days of incubation, without antibiotic therapy), and sterile histopathology. TTE and contrast-enhanced CT clearly demonstrated vegetations on the tricuspid valve. The patient denied having fever or other symptoms suggestive of infective endocarditis. Both blood cultures and histopathologic examination of the excised valve tissue revealed no evidence of an infectious process, these results were considered inconsistent with infective endocarditis according to the modified Duke criteria. In addition to valvular vegetations, imaging revealed multiple thrombi in the mesenteric, portal, and splenic veins—a pattern of venous thrombosis that is atypical for NBTE, which more commonly presents with arterial emboli. The patient had no evidence of malignancy or autoimmune disease, including negative testing for APS. However, a strong family history of thrombotic disorders raised concern for an underlying inherited thrombophilia. Conventional thrombophilia screening yielded normal results, hence subsequent genetic testing was performed and identified a pathogenic variant in the F2 gene. Notably, this mutation differed from the well-characterized G20210A variant typically associated with inherited thrombophilia, suggesting a rare or previously unrecognized prothrombotic variant. Furthermore, review of the patient's coagulation profile before anticoagulant therapy revealed a prolonged prothrombin time, decreased prothrombin activity, and reduced factor II activity (Supplementary S5). These findings are generally associated with a bleeding tendency, which appears to contradict the patient's clinical presentation of extensive thrombosis. This paradox may be explained by the functional effect of the F2 c.1621C > T (p.R541W) variant, which has been reported to impair thrombin-mediated activation of the protein C pathway, thereby shifting thrombin function toward a procoagulant phenotype. These findings are generally associated with a bleeding tendency, which appears to contradict the patient's clinical presentation of extensive thrombus formation. This paradox may be explained by antithrombin resistance (ATR). ATR refers to a condition in which thrombin or other clotting factors become less sensitive to inhibition by AT due to structural or functional changes, weakening the body's anticoagulant defenses and increases the risk of thrombosis. F2 Arg596 variants, including Yukuhashi (F2 c.1787G > T) (16), Belgrade (F2 c.1787G > A) (17), and Padua 2 (F2 c.1786C > T) (18), were reported as ATR previously. It may also be explained by its influence on activation of PC. Wu's team identified a novel F2 gene mutation, F2 c.1621C > T, in a Chinese population. This mutation moderately reduces the procoagulant activity of prothrombin and has minimal impact on its inactivation by antithrombin. However, it markedly impairs thrombin-mediated activation of protein C, both in the presence and absence of soluble thrombomodulin, thereby shifting thrombin function toward a procoagulant phenotype and ultimately increasing the risk of thrombosis (19).

For pediatric cardiology practice, this case is notable because NBTE is exceptionally rare in children and adolescents, and the published pediatric literature remains limited largely to isolated case reports and small series. Unlike the adult literature, which is dominated by malignancy- and autoimmune-associated NBTE, pediatric presentations are less well characterized and may require broader evaluation for congenital, inflammatory, hematologic, and inherited prothrombotic contributors. Accordingly, management in children should emphasize early echocardiographic recognition, rigorous exclusion of infective endocarditis, investigation of underlying thrombophilia, and individualized decisions regarding anticoagulation and surgical intervention within a multidisciplinary framework (20, 21).

Taken together, these findings support the conclusion that the hypercoagulable state driven by this rare *F2* mutation contributed to the development of both NBTE and widespread thrombosis. Meanwhile, the patient's abdominal pain was attributed to thrombosis of the mesenteric vein, as the symptom improved following anticoagulant therapy.

In this case, the patient initially presented with abdominal pain, a nonspecific symptom that posed a diagnostic challenge. Crucially, transthoracic echocardiography revealed vegetations on the tricuspid valve, enabling timely diagnosis and intervention. What makes this case particularly unusual is the involvement of the tricuspid valve in NBTE, accompanied by extensive venous thrombosis—a pattern not typically observed—and a pathogenic variant in the *F2* gene that has rarely been reported in prior studies of inherited thrombophilia.

## Conclusion

In this case the atypical presentation, including abdominal pain as the leading symptom and the absence of classical features of infective endocarditis, made early recognition of NBTE and its underlying cause especially difficult. A thorough family history ultimately prompted genetic testing, which proved critical for identifying the underlying prothrombotic condition. Thus, careful history-taking played a pivotal role in the diagnostic process and guided subsequent management.

## Ethical statement

Patient consent was obtained prior to submission of this case report. Written informed consent was obtained from the parents of the pediatric patients. The healthcare professionals explained the study details, procedures, potential risks, and benefits thoroughly to the guardians, ensuring they understood all aspects before providing consent. Institutional ethical committee approval was not obtained as it is a single case report and all patient identifiers are removed from text and figures.
