# Supplementary material for: Case Report: Non-bacterial thrombotic endocarditis and multiple thrombi uncover a hidden prothrombotic mutation
Source: Front Cardiovasc Med. 2026 Jul 7;13:1775477. doi: 10.3389/fcvm.2026.1775477 (PMC13386397; doi:10.3389/fcvm.2026.1775477)
Supplement: Supplementary file 1 [file Datasheet1.pdf]

Supplementary 1 Coagulation-related and autoantibody tests.

Test Item	Abbreviation	Result	Unit	Reference Range
Activated Protein C Resistance	APC - R	2.8	-	>2.1
Protein C	P - C	76	%	70 - 140%
Protein S	P - S	79	%	76 - 135%
Lupus Anticoagulant	LA	1.05	-	≤1.20
Anti - Phosphatidylserine/Prothrombin IgM	aPS/PT - IgM	28.7	U	≤30.0 U
Anti - Phosphatidylserine/Prothrombin IgG	aPS/PT - IgG	<9.4	U	≤30.0 U
Anti - Neutrophil Cytoplasmic Antibody (IgG Type)	IF - ANCA	Negative (-)	-	<1:10
c - ANCA - IgG	cANCA - IgG	Negative (-)	-	<1:10
Anti - Proteinase 3 Antibody (IgG Type) (CLIA)	PR3 - ANCA (CLIA)	Negative (-) <2.0	AU/mL	Negative <16.0 AU/mL; Suspected 16.0 - <24.0 AU/mL; Positive ≥24.0 AU/mL
Anti - Myeloperoxidase Antibody (IgG Type) (CLIA)	MPO - ANCA(CLIA)	Negative (-) 1.8	AU/mL	
Anti - Endothelial Cell Antibody	AECA	Negative (-)	-	<1:100

Supplementary 2 Antiphospholipid antibody profile.

**Test Item**	**Abbreviation**	**Result**	**Unit**	**Reference Range**
Anti - Cardiolipin Antibody IgG	ACL - IgG	Negative (-) 1.5	GPLU/ml	Negative <8.0 GPLU/ml;
Anti - Cardiolipin Antibody IgM	ACL - IgM	Negative (-) <2.0	MPLU/ml	Suspected 8.0 - <12.0 GPLU/ml;
Anti - Cardiolipin Antibody IgA	ACL - IgA	Negative (-) <2.5	APLU/ml	Positive ≥12.0 GPLU/ml
Anti - β2 - Glycoprotein 1 - IgG	B2GP1 - IgG	Negative (-) <2.0	AU/ml	Negative <16.0 AU/ml;
Anti - β2 - Glycoprotein 1 - IgM	B2GP1 - IgM	Negative (-) <2.0	AU/ml	Suspected 16.0 - <24.0 AU/ml;
Anti - β2 - Glycoprotein 1 - IgA	B2GP1 - IgA	Negative (-) <2.0	AU/ml	Positive ≥24.0 AU/ml

Supplementary 3 Genetic test results of the proband and his father for the *F2* gene.

Gene (Group)	Chromosomal Position	Variant Information	Zygosity Status	ACMG Classification	Inheritance Pattern
F2	chr11:46751078	NM_000506.5:exon12:c.1621C>T(p.R541W)	Heterozygous	Likely Pathogenic	AD/AR/AD/Mu

Supplementary 4. Timeline of the episode of care

Time	Clinical events	Examinations and management
Day -12	Sudden onset of diffuse, dull, distending abdominal pain. No fever, chills, syncope, diarrhea, hematochezia, palpitations, or dyspnea.	Initially self-treated with herbal remedies and later received a proton pump inhibitor at a local hospital, but symptoms persisted.
Initial outside-hospital evaluation	Persistent abdominal pain.	Contrast-enhanced abdominal CT revealed extensive thrombosis involving the mesenteric, splenic, and portal venous systems. Chest CT showed bilateral segmental pulmonary emboli in the lower lobes.
Day -4	Ongoing symptoms prompted further cardiovascular evaluation.	Transthoracic echocardiography revealed a 45-mm mobile mass on the tricuspid valve, with severe tricuspid regurgitation and right ventricular dilation. Blood cultures were obtained and remained negative after 7 days of incubation.
Preoperative period	Referred to a tertiary center for further evaluation and treatment.	Repeat CT pulmonary angiography confirmed bilateral pulmonary emboli and the tricuspid valve mass. Multidisciplinary assessment was performed.
Day 0 (Surgery)	Surgical treatment was undertaken because of the large tricuspid vegetation, severe tricuspid regurgitation, and concern for ongoing thromboembolic risk.	Surgical excision of the vegetation and tricuspid valve repair were performed under cardiopulmonary bypass.
Postoperative period	Recovery was uneventful, and abdominal pain gradually improved.	Histopathological examination of the excised vegetation showed fibrin-rich sterile thrombotic material without microorganisms. Anticoagulation therapy was initiated.
After discharge	Long-term secondary prevention and etiologic	Warfarin was prescribed for long-term anticoagulation.

	evaluation were continued.	Genetic analysis identified the F2 c.1621C>T variant in both the patient and his father.
1-year follow-up	Favorable clinical outcome without recurrent thrombotic events.	Follow-up imaging demonstrated recanalization of the portal and splenic veins.

Supplementary 5 Thrombin-related parameters before anticoagulant therapy.

Abbreviation	Test Name	Result	Reference Range	Unit
PT	Prothrombin Time	16.6	10.4 - 12.6	s
PT%	Prothrombin Activity	53.1	74.0 - 120.0	%
F Ⅱ	Factor Ⅱ Activity	35.9	79.0 - 131.0	%
